# Student Behavior Recognition From Heterogeneous View Perception in Class Based on 3-D Multiscale Residual Dense Network for the Analysis of Case Teaching

**DOI:** 10.3389/fnbot.2021.675827

**Published:** 2021-07-29

**Authors:** Hui Liu, Yang Liu, Ran Zhang, Xia Wu

**Affiliations:** ^1^School of Software Technology, Kaifaqu Campus of Dalian University of Technology, Dalian, China; ^2^Faculty of Business and Management, University Teknologi Majlis Amanah Rakyat (MARA) Sarawak Branch, Kota Samarahan, Malaysia; ^3^International School of Shenyang Jianzhu University, Shenyang, China; ^4^Huawei Nanjing Research and Development Center, Nanjing, China

**Keywords:** students behavior analysis, 3-D multiscale residual dense network, hierarchical feature, transfer learning, heterogeneous view perception

## Abstract

The study of student behavior analysis in class plays a key role in teaching and educational reforms that can help the university to find an effective way to improve students' learning efficiency and innovation ability. It is also one of the effective ways to cultivate innovative talents. The traditional behavior recognition methods have many disadvantages, such as poor robustness and low efficiency. From a heterogeneous view perception point of view, it introduces the students' behavior recognition. Therefore, we propose a 3-D multiscale residual dense network from heterogeneous view perception for analysis of student behavior recognition in class. First, the proposed method adopts 3-D multiscale residual dense blocks as the basic module of the network, and the module extracts the hierarchical features of students' behavior through the densely connected convolutional layer. Second, the local dense feature of student behavior is to learn adaptively. Third, the residual connection module is used to improve the training efficiency. Finally, experimental results show that the proposed algorithm has good robustness and transfer learning ability compared with the state-of-the-art behavior recognition algorithms, and it can effectively handle multiple video behavior recognition tasks. The design of an intelligent human behavior recognition algorithm has great practical significance to analyze the learning and teaching of students in the class.

## Introduction

A country is prosperous and strong when the education is strong. China has always attached great importance to the development of education. In the report to the 19th National Congress of the COMMUNIST Party of China (CPC), it was clearly stated that “priority should be given to the development of education.”

Over the years, the construction of educational informatization has supported and led the modernization of China's education. It has also effectively promoted the renewal of educational ideas, mode reform and system reconstruction.

With the wide application of new information technologies, such as big data, internet of things and mobile internet, university informatization has gone from “digital” to “intelligent” and entered into a new stage of the smart campus where big data, as the key supporting technology of smart campus construction, plays an irreplaceable role in smart campus construction. Big data refers to those data sets that are too large for traditional software tools to collect, store, and analyze. They are characterized by “4Vs” (namely volume, variety, velocity, and value). All kinds of system data built by the smart campus are gathered into the big data exchange platform, and massive heterogeneous multidimensional campus data are accessed, shared, distributed, and mined.

Through the comprehensive analysis of big data, the behavior rules of teachers and students in the campus and the overall operation level of the school can be grasped. The overall research and judgment and dynamic monitoring of the overall teaching and scientific research situation and development trend of the school can be carried out to transform from passive response to active service so as to realize source discovery and intelligent service.

Video understanding is a challenging task in the field of computer vision (Wang et al., 2020a; Zeng, 2020). The recognition of human behavior in video is an important branch. With the development of computer science and technology, remarkable progress has been made in the related areas. According to different ways of extracting features from video sequences, behavior recognition methods proposed in reference (Yao et al., 2019) could be divided into two categories: manual feature construction and feature automatic learning. In early human behavior recognition algorithms, manually constructed features are usually used to describe local spatial-temporal changes in videos, such as scale-invariant feature transform (SIFT) (Yin et al., 2019, 2020), histogram of oriented gradients (HOG)/histogram of oriented optical flow (HOF), motion boundary histogram (MBH) (Li et al., 2018), contour (Teng et al., 2020), motion attributes, and dense trajectory characteristics (Li et al., 2017; Papakonstantis and Tsatsara, 2018), etc. Reference (Zhang et al., 2013) proposes an improved dense trajectory (IDT) for behavior recognition; it uses the Fisher vector feature encoding, which presents excellent performance in behavior recognition. The manually constructed features are usually modeled based on human visual features and other prior knowledge to design the features. It is mainly designed for a specific task and not suitable for all scenarios. Also, its computation is complex.

With the rise of deep learning algorithms, the way of automatic learning features gradually replaces the traditional elaborately designed features. Meanwhile, the model with automatic learning is applicable to the current task. Also, the network can be trained end-to-end, which makes the model calculation more efficient. In the numerous deep learning network structures, the convolutional neural network (CNN) is the most widely used (Sun et al., 2019; Teng and Li, 2019; Yin and Bi, 2019).

CNN has achieved great success in the static image field. It also has great advantages in studying video processing. To encode spatial and temporal information in the deep convolution model, Park and Kim (2019) carries out simple and effective expansion for a 2-D convolutional network and proposes a 3-D CNN model for learning dynamic continuous video sequences and deeply learned spatial-temporal features. Li et al. (2019) finds the optimal convolution kernel size in the 3-D CNN after systematic research and proposes a 3-D CNN (C3D) that is suitable for large-scale data sets. C3D was used to extract the spatial-temporal features of the video. The extracted features had strong universality and high computational efficiency. In addition, Zhu et al. (2019) improves the 3-D CNN in the deep residual network and proposes the Res3D network, which is superior to C3D in operation speed and recognition accuracy. Hara et al. (2018) shows that the kinetics data set has sufficient data for training of deep 3-D CNN, and enables the training of up to 152 ResNets layers. Kinetics pretrains simple 3-D architectures that outperform complex 2-D architectures. Tran et al. (2018) further decomposes the 3-D convolution operations in the 3-D convolutional network into two independent continuous operations: two-dimensional space convolution and one-dimensional time convolution and proposes the R(2+1)D network. Compared with C3D, this network effectively increases the capacity of the model and is beneficial to the optimization of the network. The 3-D convolution network has received widespread attention and application because of its simple and effective strategy, but the network also has some defects. Due to its huge network parameters, convergence is very difficult. Chen et al. (2018) proposes a lightweight multifiber network architecture, which significantly reduces the computational complexity of 3-D networks and improves the model recognition performance. Yang et al. (2019) proposes an asymmetric 3-D CNN model. It reduces the number of parameters and calculation costs. This model introduces mult-source enhanced input and a multiscale 3-D convolution branch to process the convolution features of different scales in the videos. By fusing the effective information of RGB and optical flow frame, the expression ability of the model is significantly improved. The above methods only consider the depth of CNN layers; they ignore the parameter size and the time computing complexity.

Additionally, the 3-D convolution network has a gap with the baseline method in the space–time feature modeling. In the ResNext framework, the spatio-temporal channel correlation (STC) block is used as its new residual module, which could effectively capture the spatial and temporal channel correlation information in the entire network layer. Hussein et al. (2019) focuses on the time clues in behavior recognition. To model complex actions within the long time range, an improved 3-D convolutional network Timeception layer is proposed. It uses multiscale time convolution, which is learning about long-term dependencies by focusing on short-term details.

To supplement the modeling of video temporal dimension information, two CNNs were used to study the features of the original single-frame RGB image and the optical flow image of the video frame, respectively (Simonyan and Zisserman, 2014; Liu and Yin, 2017). Finally, information fusion was conducted for the output, and a dual-stream network architecture was designed to study the space–time characteristics. Wang et al. (2019) combine the sparse time sampling strategy and video fragment fusion and introduce the temporal segment network (TSN) to improve the long-term time structure modeling capability of the video. Other studies focus on using correlations between temporal and spatial networks to improve recognition performance. For example, Feichtenhofer et al. (2017) use a residual connection to conduct spatial-temporal interaction between dual-stream networks. Feichtenhofer et al. (2016) introduce fusion information into the model and specifically analyze the influence of different fusion features on the recognition results. Carreira and Zisserman (2017) extends the kernels of convolution and pooling in the double-flow network as a 3-D form and proposes an inflated 3-D ConvNet model. Through the pretraining in large data sets, it achieves advanced results (recognition rate is 97.9% in UCF-101; the recognition rate is 80.7% in HMDB-51).

Dual-flow networks and their derived models form a strong baseline. However, their application is limited due to the calculation complexity and dense sampling strategy. Ma et al. (2016) proposes a long short-term memory (LSTM) network to model the video long sequence structure. Donahue et al. (2017) proposes a long time sequence recursive neural network combining a convolutional layer and a long time sequence recursion, which could be used to learn variable length input and simulate a complex dynamic time sequence. Ng et al. (2015) computes the global video-level features by connecting multiple stacked LSTM on each frame's convolution feature. Ji et al. (2016) shows that modeling multiple flows through LSTM could improve the performance of behavior recognition. Schuldt et al. (2004) proposes that LSTM networks are able to model long-time video and high-level motion changes, but they are unable to capture important low-level movements. Furthermore, network training is time-consuming.

This paper focuses on the behavior recognition architecture based on a 3-D CNN. 3-D convolution can be used to extract universal and reliable space-time features directly from the original video, which is intuitive and effective. However, the traditional 3-D CNN algorithm lacks the full utilization of the multilayer convolution features of the network, which affects the generalization performance of the network. Combining the multiscale residual dense network and dense network, this paper proposes a 3-D multiscale residual dense network (3D-MRDN). Our main contributions are as follows:

1. This new network can make full use of the hierarchical features of all convolutional layers and uses a 3-D multiscale residual dense block (3D-MRDB) as the building module. The features of each convolutional layer in the 3D-MRDB can be transferred directly to all subsequent layers.
2. Then, local dense feature aggregation is used to retain the useful information adaptively, and local residual learning is carried out for the input and output feature aggregation. The sampled output of the 3D-MRDB module is directly accessed to all layers in the next 3D-MRDB module, forming a state of continuous transmission and feature reuse.
3. Meanwhile, the feature output in each 3D-MRDB module is used by concatenating after convolutional sampling so that multiple levels of features can be adaptively retained in a global manner to complete global feature aggregation.
4. To verify the effectiveness of the proposed algorithm, this paper trains and tests it on KTH and UCF-101 data sets. Compared with the state-of-the-art algorithms, the proposed method achieves a more accurate recognition rate. The experimental results show that the 3-D multiscale residual dense network can effectively recognize student behavior in the video.

This paper is organized as follows. In section (2), we state in detail the proposed behavior recognition framework. Section (3) gives the experiments and analysis. There is a conclusion in section (4).

## Proposed Behavior Recognition Framework

### 3-D CNN

In a 3-D CNN, 3-D convolution essentially executes the 3-D convolution kernel operation on a cube formed by stacking multiple video frames. Because each feature map in the convolutional layer is connected to multiple adjacent continuous frames in the upper layer, movement information can be captured (Scherpf et al., 2020; Ruan and Li, 2021). A 3-D convolution operation can be described as C (n, d, f), which means that it inputs the convolution layer with the size of n × n × n and d feature graphs with the size of f × f × f. The output at position (x, y, z) on the mth feature graph of the 3-D convolutional layer can be expressed as:

(1) $$\begin{array}{r} v_{lm}^{xyz}=b_{lm}+\underset{q}{\sum }\overset{f-1}{\underset{i=0}{\sum }}\overset{f-1}{\underset{j=0}{\sum }}\overset{f-1}{\underset{k=0}{\sum }}w_{lmq}^{ijk}\;v_{\left(l-1\right)q}^{\left(x+i\right)\left(y+j\right)\left(z+k\right)}, \end{array}$$

where *b*_*lm*_ is the bias of feature mapping, *q* is the feature map in the traversed *l*−1 layer, and $w_{lmq}^{ijk}$ is the weight at the kernel location q of the (i, j, k) th feature map. Weights and deviations are obtained through training.

C3D based on 3-D convolution construction is widely used in the field of video behavior recognition. Features extracted by C3D also have strong recognition ability in other tasks, such as behavior recognition, timing behavior detection, gesture recognition, etc. (He et al., 2017). Compared with C3D, improved 3-D convolutional networks based on ResNet and DenseNet architectures, such as 3D-ResNet and 3D-DenseNet, can significantly improve the effect of video behavior recognition tasks. The following are the networks constructed based on 3-D convolution. They are C3D, 3DResNet network, and 3D-MDenseNet. The input, output, and convolution kernel size of the network are the 3-D tensors with L × H × W, where L, H, and W represent the length, height, and width of time, respectively. To reduce data redundancy, even frames are skipped in the network input with size 8 × 112 × 112, which adapts to the GPU memory limit and retains an appropriate batch size. Additionally, the three networks adopt the same data enhancement and data preprocessing methods. The proposed C3D in this paper has five convolutional layers and five lower sampling layers with size 3 × 3 × 3. These are cascaded connections between layers. Finally, it goes through two fully connected layers and the Softmax layer. Its output consists of 101 category probabilities.

The 3D-ResNet network is extended by 2D-ResNet; the convolutional layer is expanded from d × d to 3 × d × d. The step of the lower sampling layer except the Conv1 convolutional layer is changed to 2 × 2 × 2. The convolution kernel of the Conv1 layer is 3 × 7 × 7, and the convolution kernel of the other layers is 3 × 3 × 3. Residual connections are adopted in the Conv1, Conv2-x, conv3-x, Conv4-x, and Conv5-x layers, which makes it easier to optimize the network. The 3-D densenet network is constructed in a similar way to the 3D-Resnet network. The hierarchical connection mode of each convolutional layer adopts a dense connection, and the network is composed of multiple dense blocks. Each layer in the same dense block reads information from all preceding layers, and finally, it is concatenated. In the same dense block, the bottleneck layer is used in which a 1 × 1 × 1 convolution operation is used to reduce the number of input feature graphs. It reduces the computation and merges the features of each channel.

### 3-D Multiscale Residual Dense Network

**Figure 1:**
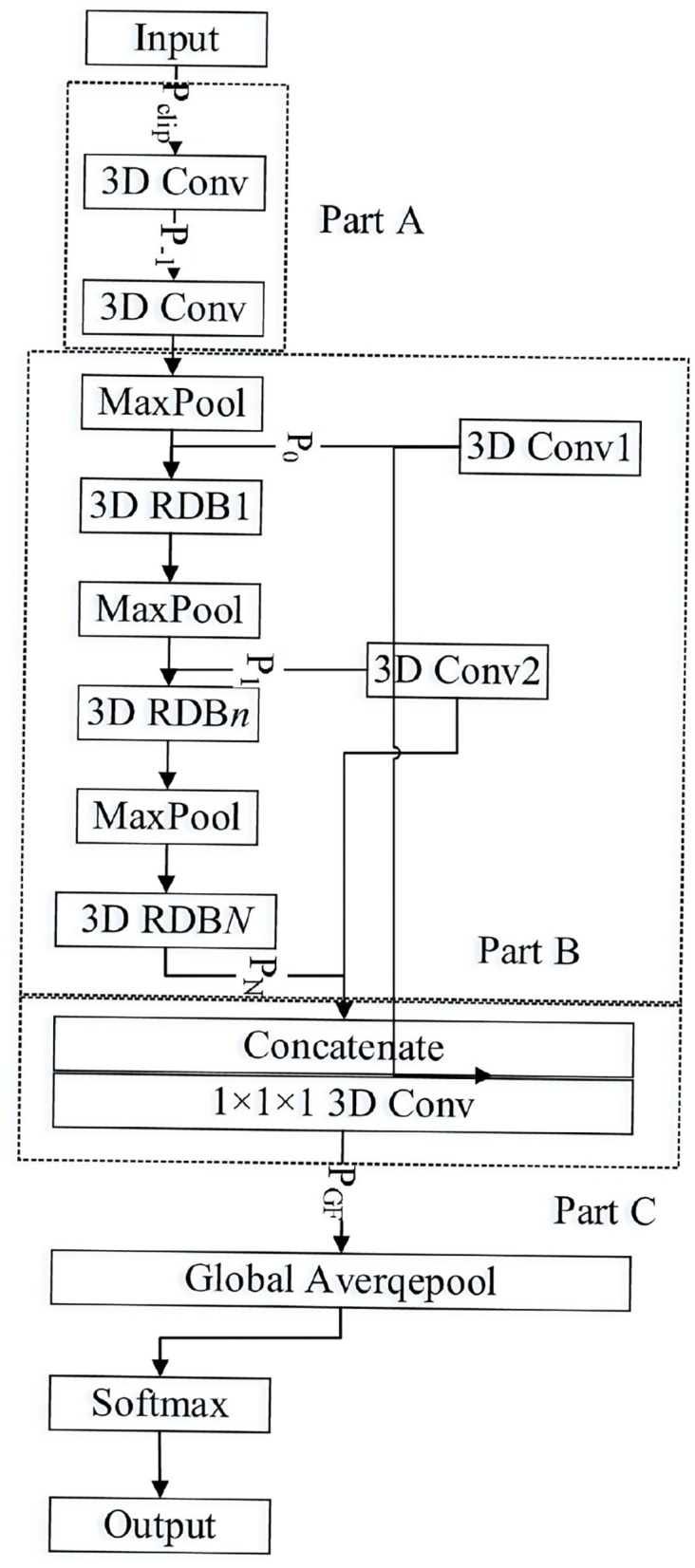
3D multiscale dense residual network.

The proposed 3-D multiscale residual dense network (3D-MRDNet) imitates the residual of ResNet learning and the dense DenseNet network connection mode to build the 3-D residual dense blocks and extract the multilevel space–time features in 3-D video behavior recognition. It combines low-level features with high-level semantic features to improve the expression ability of the model as shown in Figure 1.

As shown in Figure 1, the 3-D multiscale dense residual network is divided into three parts: the shallow feature extraction layer, the multiscale dense residual layer, and the global feature aggregation layer (Wang et al., 2020b). The shallow feature extraction layer (Part A) includes the two 3-D Conv, and the multiscale residual dense layer (Part B) includes a pooling layer (Maxpool), multiple residual dense blocks (3D-RDB), and convolutional layer 3D Conv1 and 3D Conv2 for convolution down-sampling. The global feature aggregation layer (Part C) consists of a concatenated layer for feature splicing and a convolutional layer for feature aggregation.

The input and output of the 3-D residual dense network are defined as *P*_*clip*_ and *P*_*cls*_, respectively. The first two convolutional layers of the network are used to extract shallow features. Specifically, the process of extracting features from the shallow layer can be described as

(2) $$\begin{array}{r} P_{0}=G_{sh}\left(P_{clip}\right), \end{array}$$

where *G*_*sh*_ represents the composite function of the first two convolution layers and the down-sampling operation, and *P*_0_ is the feature graph extracted from the video clip, which is used for input of the first layer residue dense block. Here, *N* residual dense blocks are set. The output of the nth residual dense block is *P*_*n*_. Its calculation process is as follows:

(3) $$\begin{array}{l} P_{n}\text{= }G_{3}D-MRDB,n(GD-MRDB,-1\\ \;(\cdots (G_{3D-MRDB,1}(P_{0}))\cdots )), \end{array}$$

where *G*_3*D*−*MRDB, n*_ represents the calculation operation of the nth residual dense block (3D-MRDB) and sub-sampling (Maxpool). When *n* = *N*, *G*_3*D*−*MRDB, N*_ only contains the computation operation of the residual dense block. *G*_3*D*−*MRDB, n*_ is a composite operation function that includes multilayer convolution and rectifying linear units. Because *P*_*n*_ is generated by multiple convolution layer operations in the nth residual dense block, *P*_*n*_ can be regarded as a locally dense feature.

After multilayered local dense features are extracted by 3D-MRDNet through multiple 3D-MRdB, global feature aggregation (GFA) is further achieved. The GFA takes full advantage of the features in the preceding layers. Specifically, the feature *P*_*n*_ in different levels of input is sampled as a 1 × 7 × 7 feature graph *X*_*n*_. And *l*_2_ norm normalization is performed. Then, concatenate is used to splice the local dense feature *X*_*n*_ from different levels. The convolution with size 1 × 1 × 1 is used for feature aggregation and channel adjustment to obtain the feature graph of global feature aggregation where the stitching process of local dense features can be described as follows:

(4) $$\begin{array}{r} P_{GFA}=G_{GFA}\left(\left[X_{0},X_{1},\cdots \;,X_{N}\right]\right), \end{array}$$

where *P*_*GFA*_ is a feature graph output by the global feature aggregation. *G*_*GFA*_ is a composite function with 1 × 1 × 1 convolution, which is used for features adaptive fusion from different layers. [*X*_0_, *X*_1_, ⋯ , *X*_*N*_] refers to the concatenation of N feature graphs after 3-D residual dense blocks and convolution sampling.

Based on the above analysis, the network extracts shallow features from the input clip, and then it obtains rich local features through multiple residual dense blocks and gets global features through global feature aggregation. Finally, it obtains scores of each class through a softmax classifier. The entire network 3D-MRDNet calculation process can be expressed as

(5) $$\begin{array}{r} P_{cls}=G_{MRDNet}\left(P_{clip}\right), \end{array}$$

where *G*_*MRDNet*_ is the operation of the entire 3D-RDNet network. *P*_*cls*_ is the output of the network.

### 3-D Residual Dense Block

**Figure 2:**
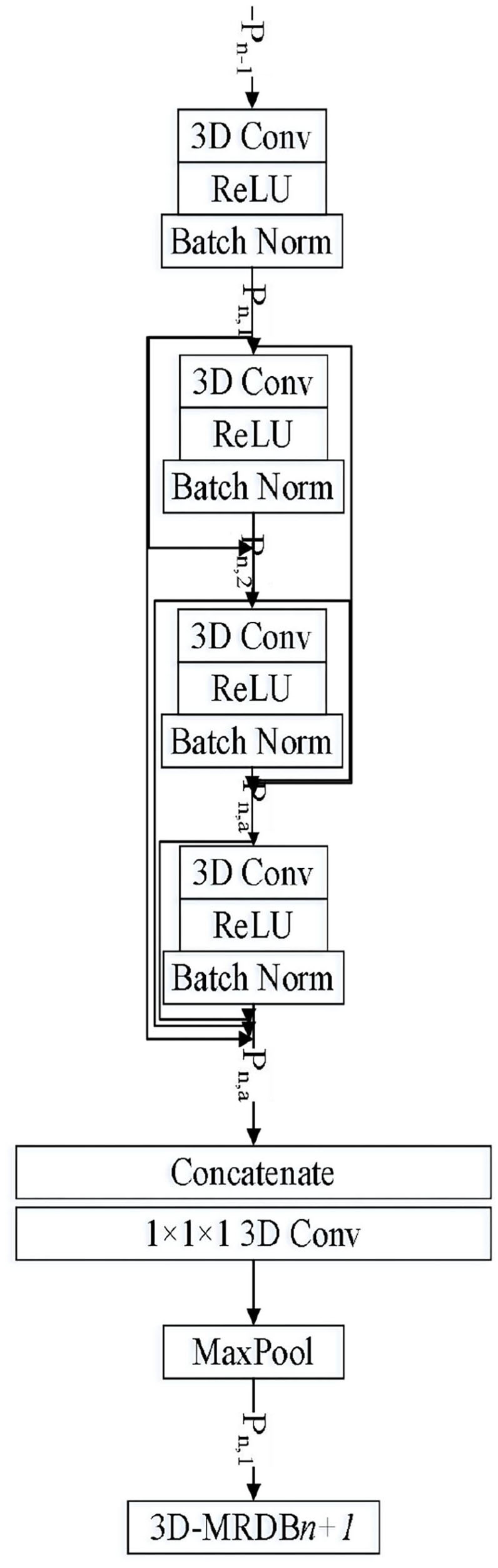
3-D residual sense block.

A 3-D residual dense network is composed of multiple 3-D residual dense blocks. Figure 2 is the network structure diagram of 3-D residual dense blocks (3D-RDB). 3D-RDB includes dense joint layers, local feature aggregation (LFA), and local residual learning (LRL), which enables the network to fully learn multilayer convolution features.

The 3D-RDB module consists of multiple convolution layers, a linear rectifier unit (ReLU), and the batch normalization (BatchNorm) feature extraction unit is beneficial for training of the deeper network. The features learned by the former 3D-RDB are delivered directly to each layer in the current 3D-RDB. Meanwhile, there is a direct connection between each layer inside the module, which makes the transfer of features and gradients more effective. It promotes feature reuse, retains the forward-propagating feature, and extracts the local dense feature. Here, we define *P*_*n*−1_ and *P*_*n*_ as the nth input and *n* + 1th input of 3D-RDB, respectively. The output of the *a*th Conv layer in the nth 3D-RDB can be expressed as

(6) $$\begin{array}{r} P_{n,a}=\sigma \left(W_{n,a}\left[P_{n-1},P_{n,1},P_{n,2},\cdots \;,P_{n,a-1}\right]\right), \end{array}$$

where σ is the activation function of ReLU, *W*_*n, a*_ is the weight of the *a*th convolution layer, and the bias term is omitted here for simplicity.

Assuming that *P*_*n, a*_ is composed of a multiple feature graph, [*P*_*n*−1_, *P*_*n*, 1_, *P*_*n*, 2_, ⋯ , *P*_*n, a*−1_, ] is the concatenation of the output feature graph of the (*n*−1) th 3D-RDB and the nth 3D-RDB.

After learning multilevel spatiotemporal features through the dense connection mode, 3D-RDB fuses local dense features. Specifically, a series of convolutional layer features from the previous 3D-RDB and the current 3D-RDB are extracted and spliced. A 1 × 1 × 1 convolutional layer is introduced for adaptive feature fusion with different levels, and this operation is named LFA. The calculation process can be described as follows:

(7) $$\begin{array}{r} P_{d,LF}=G_{LFA}^{n}\left(\left[P_{n-1},P_{n,1},P_{n,2},\cdots \;,P_{n,a},\cdots \;,P_{n,A}\right]\right), \end{array}$$

where $G_{LFA}^{n}$ represents the composite operation of the 1 × 1 × 1 convolutional layer in the nth 3D-RDB, which can reduce the number of feature graphs and the computations to fuse each channel at the same time. As the growth rate of dense networks increases, LFA contributes to very dense network training.

In the deep network structure, to ensure the maximum information flow between different levels in the network, the skip connection mode of the residual network is adopted in 3D-RDB, which connects feature graphs with the same feature map size so that the output of each layer is directly connected to the input of the subsequent layer. This kind of jumping connection alleviates the problem of network gradient disappearance, enhances feature propagation, promotes feature reuse, and retains the features of forward propagation. The output of the nth 3D-RDB can be expressed as

(8) $$\begin{array}{r} P_{n}=P_{n-1}+P_{n,LF}. \end{array}$$

The use of LRL can improve the network expression ability and achieve a better network effect. This module architecture is called a 3-D residual dense block (3D-RDB) due to dense connection patterns and LRL.

In the proposed 3D-MRDNET network, the convolution kernel size is 1 × 1 × 1 in local and global feature aggregation; the convolution kernel size in other layers is set as 3 × 3 × 3. There are 96 filters in the first layer of the network, 512 filters in the GFA convolution layer, and 128 filters in the rest of the network.

In addition, the number of dense 3-D residual blocks in the 3D-RDNet network tested in this paper is set as three, and the number of dense layers within the dense 3-D residual block is set as four. In the 3D-RDNet network, except for convolutional layers, such as 3D Conv1 and 3D Conv2, which are convolved with deconvolution sampling in the dense residual layer, the other structural parameters are shown in Table 1, where the step size is 2 × 2 × 2.

**Table 1:** The parameters in 3D-MRDNet.

**Network layer**	**Size of output**	**Structure parameter**
Conv1	8 × 112 × 112	3 × 3 × 3, stride, 1 × 2 × 2
Conv2	8 × 56 × 56	3 × 3 × 3
Conv3_x	4 × 28 × 28	$\left[\begin{array}{l} 1\times 1\times 1\\ 3\times 3\times 3 \end{array}\right]$ × 4
Conv4_x	2 × 14 × 14	$\left[\begin{array}{l} 1\times 1\times 1\\ 3\times 3\times 3 \end{array}\right]$ × 4
Conv5_x	1 × 7 × 7	$\left[\begin{array}{l} 1\times 1\times 1\\ 3\times 3\times 3 \end{array}\right]$ × 4
–	1 × 1 × 1	Global average pooling, 101-d FC, softmax

Four network model parameters (Params) and floating point operations (FIOPs) can be obtained by analyzing Table 1 as shown in Table 2.

**Table 2:** Params and FIOPs in different models.

**Network**	**Params/10^**6**^**	**FIOPs/10^**9**^**
C3D	11.6	6.3
3D-RESnet	33.1	19.2
3D-Densenet	17.5	8.1
3D-MRDNET	13.5	6.8

Table 2 shows that, compared with C3D, the proposed 3D-MRDNet network has more parameters and computations. Compared with 3D-ResNet and 3D-DenseNet models, 3D-MRDNet has the advantages of fewer parameters and less computations.

## Experiments and Analysis

### Data Sets

**Figure 3:**
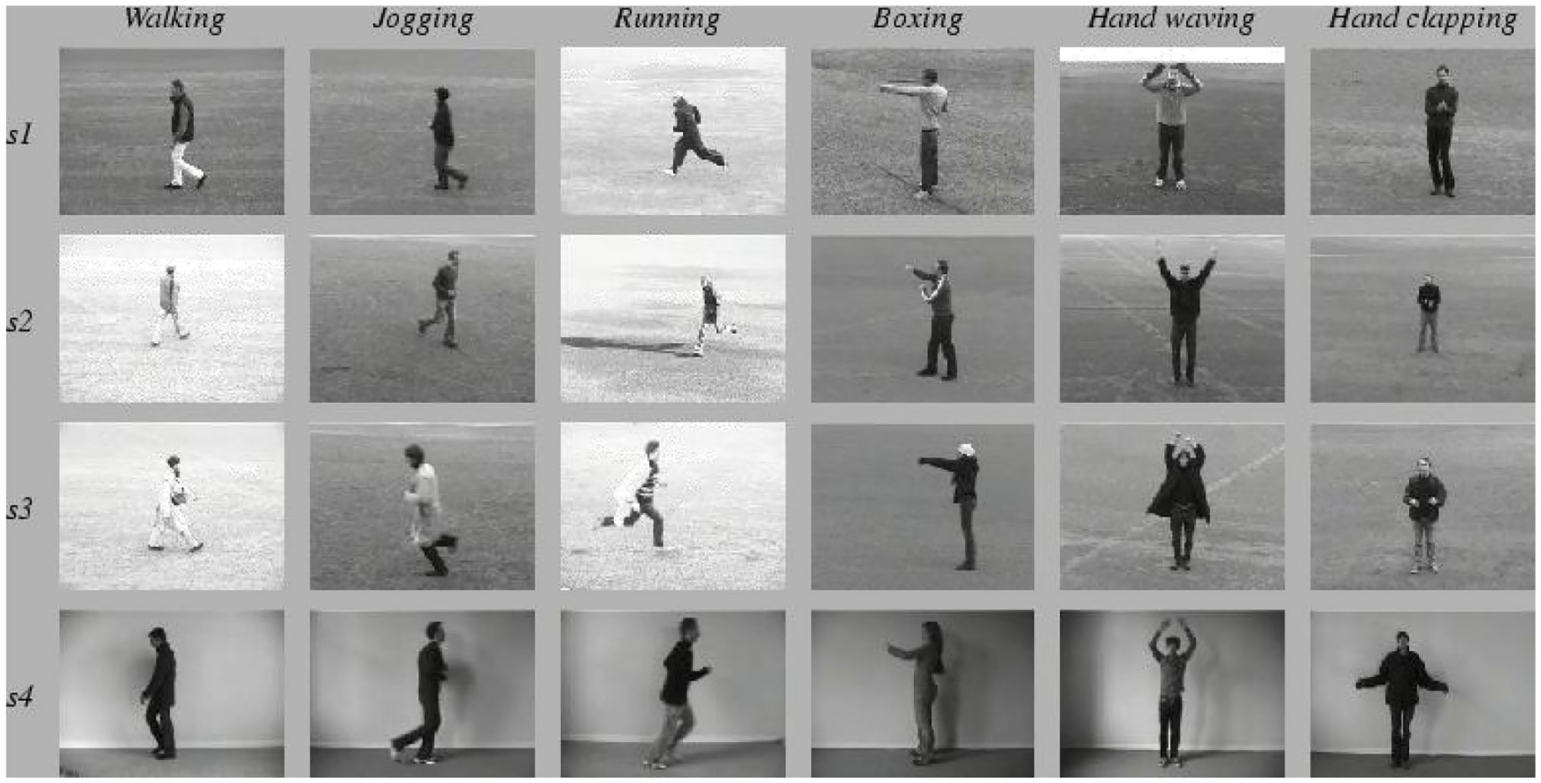
Six types of behavior examples in KTH data set.

The experimental data in this paper include KTH and UCF-101 as well as the data set collected and produced in this paper, including student behaviors in class. KTH and UCF-101 are the most commonly used data sets in the field of computer visual behavior recognition. The KTH data set is completed by 25 people performing six action types under four different scenarios with a total of 600 video samples. Here, behavioral categories include boxing, clapping, waving, jogging, running, and walking. The four scenarios include different lighting conditions, clothing changes, and background scale changes. However, its background is relatively simple with few behavioral categories, and the camera shooting angle is fixed. The experiment in this paper uses the behavioral video with 16 people as training and the behavioral video of the remaining nine people as test. The six kinds of actions in the KTH data set are shown in Figure 3.

**Figure 4:**
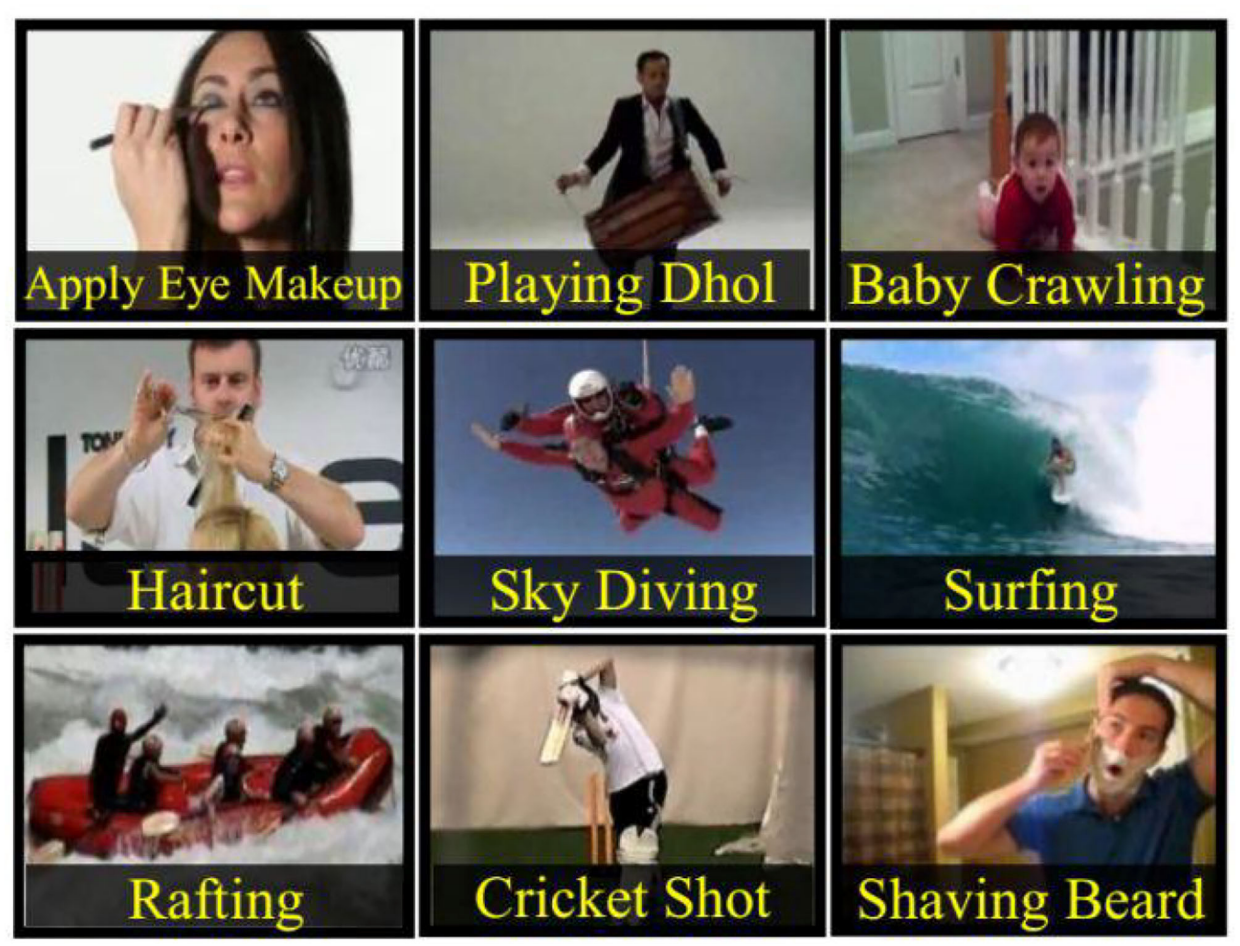
Nine types of behavior examples in UCF-101 data set.

UCF-101 is also the most widely used data set in the field of behavior recognition. The sample is collected from the YouTube video site and consists of 13,320 videos and 101 behavioral categories. UCF-101 provides a great variety of behavior categories. Different from previous data sets, it is characterized by great variation in background type, camera movement, lighting conditions, angle of view, object proportions, and posture. Videos in each behavior category are divided into 25 groups, each containing 47 behavior videos. The behavior can be divided into five types: (1) human–object interaction, (2) only physical movement, (3) human interaction, (4) play an instrument, (5) movement. There are three recommended training/test groupings for the entire data set. In this paper, the split1 training/testing group recommended by UCF-101 is used for experiments. The nine types of behavior listed by UCF-101 are shown in Figure 4.

**Figure 5:**
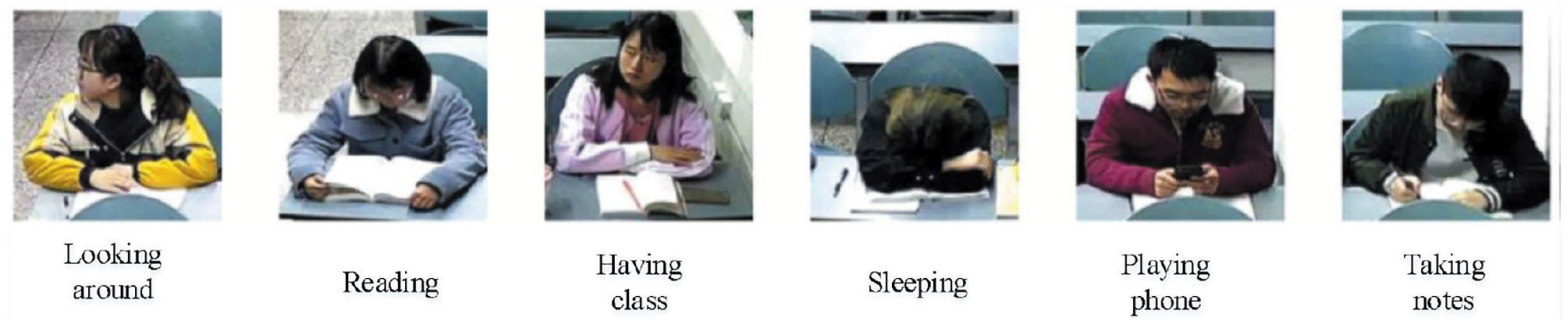
Student behavior examples in class.

In this paper, the data sets of real scenes are established according to the requirements of teaching tasks in class. The video is collected through the camera equipment installed in the classroom with a resolution of 2,560 × 1,536 and includes six actions frequently appearing in class: having class, sleeping, playing on a mobile phone, taking notes, looking around, and reading. After collecting the video, uniform frame sampling is carried out to convert the video into images, and then the original images are cropped into images containing individual students and reconstructed into 128 × 128 pixels. After labeling the class behavior of students in each image, a total of 1,020 labeled class behavior images are obtained. The original data set is expanded by mirror-symmetric data enhancement. The class behavior recognition data set containing 2,040 images is finally obtained. Some of the images in the data set are shown in Figure 5 in which the number of images for each behavior is the same; 1,560 images are randomly selected as the training set, and the remaining 480 images are used as the test set.

### Experiment Set

This paper adopts Keras (an open deep learning framework of Google). The experimental platform is Ubuntu 16.04 and NVIDIA GTX1060. The input of the model is a clip composed of 16 consecutive frames of a video sequence. The sampling rate of the clip is set to two. During the training, skipping even frames is adopted on the network input. The random cropping, random flipping, and random rotation of images are used to increase the diversity of training samples. During the test, the input video clips are preprocessed in the same way as the training stage, and then the trained model is used to estimate the behavior classification of each video clip sequence. If the classification results of the whole video level are needed, multiple clips of the current video are selected to obtain the classification results, respectively. Then the final behavior classification of the video is averaged.

### The Experiment on the KTH Data Set

In order to verify the effectiveness of the 3-D multiscale residual dense network proposed in this paper, experiments are carried out on three data sets, including KTH, UCF-101, and the real scene data set made by ourselves. First, experiments are carried out on the small KTH data set to test the video behavior recognition accuracy of four network models, respectively.

When training the data, after data enhancement and preprocessing, the video frame input size of the KTH data set is 8 × 112 × 112. Batch size is set to 16; it uses the Adam optimizer. Parameters are β_1_= 0.9, β_2_= 0.999. The initial learning rate is 10^−4^. The loss function uses a multiclass cross-entropy function, and the training duration is 25 cycles. The performance results of human behavior recognition algorithms, such as C3D, 3DResNet, 3D-DenseNet, 3D-MRDNet, DHL (Cahyadi et al., 2018), SPPDCN (Yang et al., 2018), and MMDSTL (Zhao et al., 2019) are shown in Table 3. Here, the accuracy of behavior recognition is calculated based on video-level classification results, namely video top-1. Cahyadi et al. (2018) proposes an improved recurrent neural network matching strategy by explicitly transforming the feature in Euclidean space by a distance learning function. The distance function is based on a simple Siamese network with two subnetworks sharing the same weights. The network consists of the learned feature based on unsupervised dictionary learning as an intermediate layer between raw input and fully connected layers with non-linear activation and regularization. Yang et al. (2018) proposes a 3-D densely connected convolutional network based on spatial pyramid pooling (3D-DenseNet-SPP). As the name implies, the network structure is mainly composed of three parts: 3DCNN, DenseNet, and SPPNet. These models were evaluated on a KTH data set and UCF101 data set separately, which got better results. Zhao et al. (2019) proposes a dual-stream 3-D space–time CNN action recognition framework and achieves the best result in the test on the public data set.

**Table 3:** Comparison with different methods on KTH data.

**Method**	**Accuracy**	**False detection rate**
DHL	71.52%	21.26%
SPPDCN	79.38%	18.47%
MMDSTL	88.47%	15.33%
C3D	90.27%	10.25%
3D-ResNet	91.57%	6.94%
3D-DenseNet	92.36%	5.93%
**Proposed**	**94.28%**	**1.65%**

*The bold values denote the best values with proposed method*.

**Figure 6:**
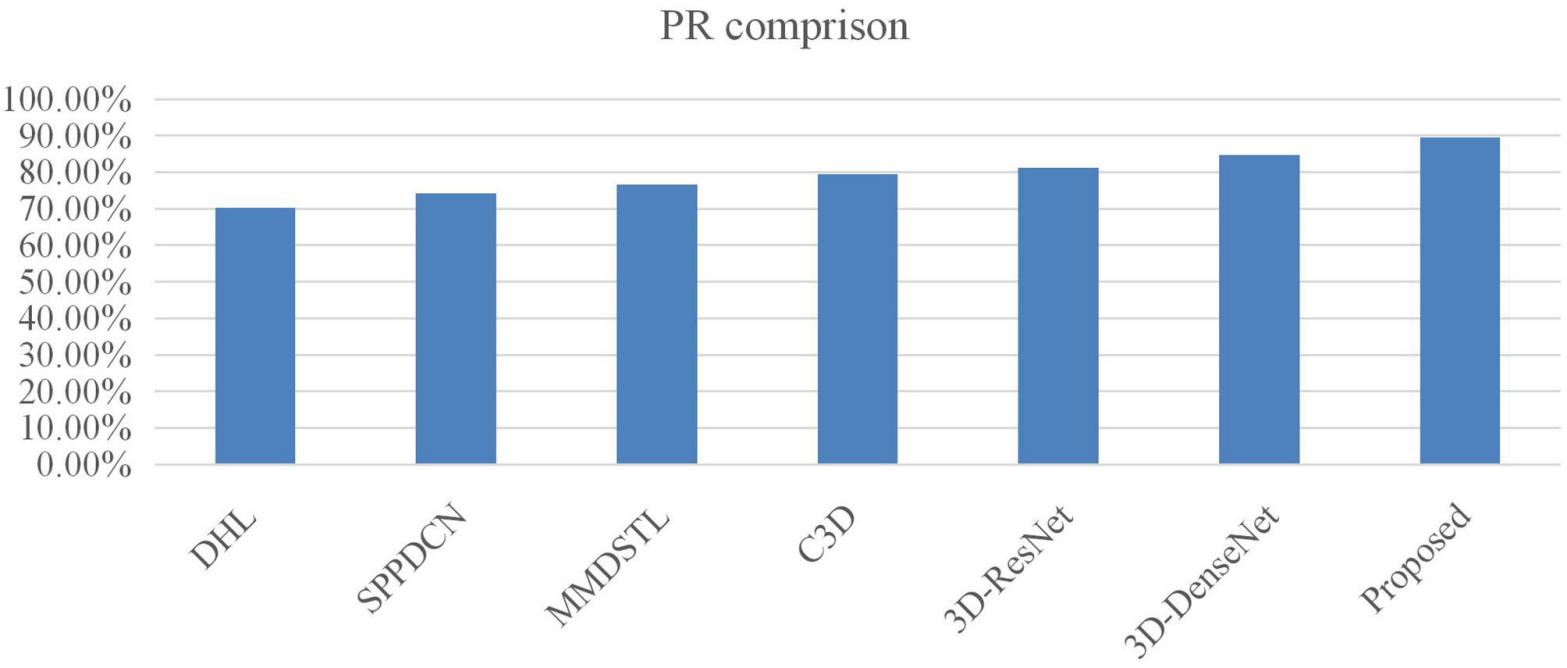
PR comparison.

It can be seen from Table 3 that the accuracy of the 3-D multiscale convolutional network in the KTH data set has a great advantage over other algorithms. Meanwhile, the improved networks 3D-ResNet and 3D-DenseNet have better results than C3D, which improved by 1.30 and 2.09% over C3D. And the proposed 3-D multiscale residual density network in this paper is improved by 4.01% over the C3D. Figure 6 is the PR comparison with the different methods. It shows that the PR with proposed method is better than other models.

The model trained on the KTH training set is tested on the whole data set, and the confusion matrix is obtained as shown in Table 4. It can be seen from the confusion matrix that the overall recognition rate of the 3D-MRDNet network on the KTH data set is very high, but the model is not very good at distinguishing running, jogging, walking, and other behaviors. On the one hand, the similarity of these actions is higher. On the other hand, the resolution of the video is low, which easily causes misjudgment. In general, the 3D-MRDNet network has a good recognition effect on the KTH data set. The trained model on the KTH training set tests the whole data set and obtains a 98.2% recognition rate.

**Table 4:** Confusion matrix with proposed method on KTH data set.

True label	Jogging	0.98	0.00	0.00	0.02	0.02	0.00
	Boxing	0.00	1.00	0.00	0.00	0.00	0.00
	Waving	0.00	0.02	0.97	0.00	0.00	0.01
	Walking	0.02	0.00	0.00	0.98	0.00	0.00
	Running	0.07	0.00	0.00	0.01	0.95	0.00
	Clapping	0.00	0.00	0.00	0.00	0.00	1.00
		Jogging	Boxing	Waving	Walking	Running	Clapping
		Predicted label					

### Experiments on UCF-101 and Real Scene Data Set

This paper also tests the UCF-101 data set and an established real scene data set. The parameters are basically the same during the experiment. The input of the network is a clip (a continuous 16 frames of a video extracted from each video). The width and height of the video frame are resized as 171 × 128. After data preprocessing, the input size is cut into 8 × 112 × 112. In the aspect of network optimization, the stochastic gradient descent method is adopted. The parameters of the network are as follows: initial learning rate = 0.01; momentum parameter = 0.9, learning decay rate = 10–4, objective function = cross-entropy loss function, network training cycle = 25, batch processing size = 16. The experimental results of four networks C3D, 3D-Resnet, 3D-Densenet, 3D-RDNET, and other human behavior recognition algorithms in UCF-101 and real scene data sets are given in Table 5. Where the accuracy is calculated based on Clip top-1, which is 16 frames in a row.

**Table 5:** Accuracy comparison with different methods on UCF-101 and real scene data.

**Method**	**UCF-101**	**Real scene data**
DHL	71.68%	79.63%
SPPDCN	72.93%	81.55%
MMDSTL	76.84%	87.37%
C3D	81.55%	89.68%
3D-ResNet	86.95%	92.01%
3D-DenseNet	91.87%	93.21%
Proposed	**96.79%**	**96.86%**

*The bold values denote the best values with proposed method*.

The following can be seen from Table 5:

1. For the UCF-101 data sets, for the proposed model (3D-MRDNet) compared with 3D-ResNet, 3D-DenseNet, and C3D, the human behavior recognition rate increased by 9.84, 4.92, and 15.24%, respectively. It shows that the 3D-MRDNet network combined with dense connection and residual learning on complex data sets is more excellent than a single network structure. The identification accuracy is superior to other methods. It verifies the effectiveness of the designed network and better performance.
2. On the real scene data set, the recognition effect of the model in this paper is also better than that of other networks, which achieves a recognition rate with 96.86%. In conclusion, the 3D-MRDNET network is still competent for tasks in real scenes, and the network has good robustness and transfer learning ability.

## Conclusions

To solve the problem that the traditional 3-D CNN algorithm lacks full utilization of the network's multilevel convolutional features, this paper proposes a 3-D multiscale residual dense network architecture for human behavior recognition and verifies the effectiveness of the proposed algorithm on public and real scene data sets. The main work of this paper is as follows:

1. The 3-D CNN is improved, and the 3-D multiscale residual dense network is proposed, which ensures the accuracy of the network and reduces the complexity of the model.
2. A network construction module (3-D multiscale residual dense block) is proposed. Through a dense connection mode, LFA, and LRL, the network's ability to fully learn multilayer convolution features is enhanced, and the loss risk of original video information during network training is reduced.
3. The proposed algorithm uses multiple 3-D residual dense blocks to extract multilevel spatio-temporal features and then combines low-level features with high-level semantic features through global feature aggregation to improve the expression ability of the model.

In addition, the data enhancement method and data preprocessing method can significantly prevent the overfitting phenomenon in the process of network training. Through a public data set and real scene experimental data set, it verifies that the proposed algorithm is better than most of the traditional algorithms as well as the 3-D convolution, which significantly enhances the accuracy in the video behavior recognition task. In future work, we will research more advanced deep learning methods for human behavior recognition.

## Data Availability Statement

The data analyzed in this study is subject to the following licenses/restrictions: the data used to support the findings of this study are available from Yang Liu, liuyang@sjzu.edu.cn. Requests to access these datasets should be directed to liuyang@sjzu.edu.cn.

## Ethics Statement

Written informed consent was obtained from the individual(s) for the publication of any potentially identifiable images or data included in this article.

## Author Contributions

HL and YL: drafting and refining the manuscript. RZ and XW: critical reading of the manuscript. All of the authors have read and approved the manuscript.

## Conflict of Interest

The authors declare that the research was conducted in the absence of any commercial or financial relationships that could be construed as a potential conflict of interest. The handling editor declared a shared affiliation, though no other collaboration, with the authors HL and RZ.

## Publisher's Note

All claims expressed in this article are solely those of the authors and do not necessarily represent those of their affiliated organizations, or those of the publisher, the editors and the reviewers. Any product that may be evaluated in this article, or claim that may be made by its manufacturer, is not guaranteed or endorsed by the publisher.
